# Precision Oncology in Clinical Practice: Two Years of Comprehensive Genomic Profiling in Croatia †

**DOI:** 10.3390/jpm15020059

**Published:** 2025-01-31

**Authors:** Dora Čerina Pavlinović, Jelena Šuto Pavičić, Antonela Njavro, Nikša Librenjak, Ilijan Tomaš, Robert Šeparović, Stjepko Pleština, Žarko Bajić, Natalija Dedić Plavetić, Eduard Vrdoljak

**Affiliations:** 1 Department of Oncology, University Hospital Center Split, 21 000 Split, Croatia; dcerina@kbsplit.hr (D.Č.P.); jsuto@kbsplit.hr (J.Š.P.); 2 School of Medicine, University of Split, 21 000 Split, Croatia; 3 Department of Oncology and Nuclear Medicine, Sestre Milosrdnice University Hospital Center, 10 000 Zagreb, Croatia; antonela.vrljicak@kbcsm.hr; 4 Department of Oncology, University Hospital Center Zagreb, 10 000 Zagreb, Croatia; niksa.librenjak@kbc-zagreb.hr (N.L.); stjepko.plestina@kbc-zagreb.hr (S.P.);; 5 School of Medicine, Josip Juraj Strossmayer University of Osijek, 31 000 Osijek, Croatia; tomas.ilijan@kbco.hr; 6 Department of Medical Oncology, University Hospital for Tumor, Sestre Milosrdnice, 10 000 Zagreb, Croatia; robert.separovic@kbcsm.hr; 7 School of Medicine, University of Zagreb, 10 000 Zagreb, Croatia; 8 Research Unit “Dr. Mirko Grmek”, Psychiatric Clinic Sveti Ivan, 10 000 Zagreb, Croatia; zarko@biometrika.hr

**Keywords:** comprehensive genomic profiling, precision oncology, real-world data

## Abstract

**Background:** The widespread adoption of precision medicine in routine cancer care remains a critical challenge, even as advanced technologies expand and personalized therapies demonstrate remarkable success in certain cancer types. While breakthrough innovations in targeted treatments have revolutionized outcomes for specific cancers, translating these scientific advances into standard clinical practice continues to be an evolving and complex endeavor. Croatia has a nationwide project of precision oncology through the comprehensive genomic profiling (CGP) analysis. Since collecting and analyzing real-world data is crucial for clinical research and defining the value of CGP in precision oncology, we aimed to present the data from everyday clinical practice given the opportunities and challenges we faced. **Methods**: This was a retrospective observational study conducted at the national level in all patients whose tumor samples were subjected to CGP between 1 January 2020 and 31 December 2021. **Results**: In total, 481 patients with CGP results were included in this study. Gastrointestinal and reproductive malignancies were the most common, accounting for 29.1% and 28.9% of all tested tumors, respectively. Specifically, colorectal tumors made up 19.1% of cases, while uterine tumors represented 11.2%. At least one clinically relevant genomic alteration was found in 76.7% of patients, with the KRAS mutation (27.2%) being the most common. During the two-year study period, 26,709 individuals lost their lives to cancer in Croatia. Combining this with the CGP selection criteria valid at the time, there was an estimated population of approximately 13,350 potentially eligible patients for the CGP analysis, meaning that only 3.6% of potentially eligible patients were tested. **Conclusions**: The analysis identified clinically actionable genomic alterations in approximately 80% of the evaluated patients, suggesting they could be candidates for targeted therapeutic interventions. The adoption of CGP remains limited, with estimates indicating that under 5% of metastatic cancer patients received testing in the initial two-year implementation period, despite established national insurance coverage guidelines. This low utilization rate suggests a significant gap in access to genomic testing, leaving many eligible cancer patients without the potential benefits of this diagnostic approach.

## 1. Introduction

Unlike traditional treatment approaches, in which one size fits all, precision medicine transforms patient care and highlights the need for diagnostic and treatment personalization. Furthermore, as one of the most rapidly evolving fields in medicine, oncology has an absolute leading role in implementing these postulates in everyday clinical and scientific practice. A personalized approach starts from the beginning, encompassing the right diagnostic procedures and interventions at the right time. Nevertheless, it also relies on patients’ general health, medical history and comorbidities, lifestyle, and environmental factors [1]. In addition, owing to our knowledge constantly expanding and being amended, the diagnosis of cancer type today goes beyond simply defining its histological type [2]. This expanded knowledge contributes to defining the correct diagnosis, as well as determining the treatment pathway. Over the last two decades, breakthroughs in molecular biology and underlying carcinogenesis have led to the development of new treatment strategies, such as immunotherapy and molecular-targeted therapy [3,4]. These cutting-edge therapies have already significantly changed outcomes in the metastatic setting of several cancer types [5,6,7,8,9,10,11]. Moreover, in addition to improving survival, they often enhance patients’ quality of life by minimizing side effects, which are usually accompanied by conventional treatment [12]. Consequently, it is mandatory to define the molecular background of the tumor at the time of diagnosis of metastatic disease, making tumor genomic profiling the backbone of precision oncology. Enhancements in the knowledge, methodology, and financial affordability of modern technologies such as next-generation sequencing by hybrid capture have enabled the introduction of comprehensive genomic profiling (CGP) into clinical practice. Several CGP assays have been approved by the U.S. Food and Drug Administration (FDA) for diagnostic, prognostic, and therapeutic purposes, one of which is FoundationOneCDx (Foundation Medicine Inc., Cambridge, MA, USA) [13,14,15]. The results of the CGP assay provide direct insight into the tumor genomic profile and potential targetable “weak” spots, making each patient’s tumor unique and providing the opportunity to tailor the treatment in accordance with the results. This approach is currently a hot topic, and, as a result, new clinical trials, such as basket and umbrella trials, have been designed [16,17]. In addition, one of the important aspects of implementing precision in practice comes from professionals in everyday work, and one of these analyses revealed that professionals perceive the use of precision medicine as an enchantment and distraction [18]. In addition, in this personalized era, the amount of information and a diverse patient population exceed the scope of clinical trials, and emphasis is placed on real-world data where one is learning from and for every patient individually [19]. Croatia stands as a pioneer in implementing nationwide precision oncology through CGP in routine clinical care, with full coverage provided by the national health insurance system. This groundbreaking initiative places Croatia at the forefront of personalized cancer treatment, making advanced genomic analysis accessible to cancer patients across the country through their standard healthcare coverage. The main goal of the project is to generate a national clinical genomic database on the bases of the findings of the CGP analysis provided by Foundation Medicine. Despite the increased availability of modern technologies and the evolution of tailored treatment, the applicability of precision medicine in everyday clinical practice is still emerging. We aimed to present data on a national level from the first two years of testing given the opportunity and challenges we faced.

## 2. Materials and Methods

### 2.1. Study Design

This nationwide observational retrospective analysis examined patients who underwent comprehensive genomic profiling of their tumor samples during the two-year period from January 2020 through December 2021. For real-world CGP data analysis, a cross-sectional study was conducted at six Croatian institutions: University Hospital Centre Split, University Hospital Center Zagreb, Sestre Milosrdnice University Hospital Centre in Zagreb and their Clinic for Tumors, and University Hospital Centers Rijeka and Osijek. The study was approved by the Ethics Committees of all the participating institutions. Informed consent was obtained from all patients before CGP analysis and data collection. The data files were anonymized before the analysis and the study was performed following the World Medical Association Declaration of Helsinki of 1975 as revised in 2013 [20]. The study was not pre-registered, nor were the data reviewed centrally.

### 2.2. Comprehensive Genomic Profiling Analysis

The CGP analysis was performed through FoundationOneCDx testing for solid tumors, FoundationOne Liquid (blood test for circulating tumor DNA for solid tumors in cases of insufficient tissue), and FoundationOne Heme for hematological malignancies and sarcomas. The genomic analysis findings are categorized into two main classifications: first, mutations with established clinical significance that can be targeted by approved therapies, experimental treatments, or ongoing clinical trials; and, second, genetic variations whose clinical impact remains undetermined.

FoundationOneCDx was used for tissue analysis. Previously inspected by pathologists from included institutions, formalin-fixed, paraffin-embedded tissue was sent as a block, with one hematoxylin-and-eosin-stained slide or 10 unstained slides and one hematoxylin-and-eosin-stained slide. After DNA extraction, samples containing 50–1000 ng underwent comprehensive genomic analysis using whole-genome shotgun library preparation and targeted capture techniques. The analysis focused on 324 genes, encompassing 309 tumor-related exons, one promoter region, one noncoding RNA, and select intronic regions from 34 genes commonly involved in tumor rearrangements. Sequencing was performed on the Illumina^®^ HiSeq 4000 platform (Illumina, Inc. San Diego, CA, USA), achieving uniform coverage with a median depth exceeding 500×, and more than 99% of exons covered at >100×. The genomic assessment included four key alteration types: base substitutions, insertions/deletions, copy number variations, and gene rearrangements. Additional analyses included the following: microsatellite status evaluation utilized 95 microsatellite loci across the genome and the tumor mutational burden (TMB) calculation incorporated both synonymous and nonsynonymous variants with ≥5% allele frequency, expressed as mutations per megabase (Muts/Mb) [15].

FoundationOne Liquid CDx analyzes cell-free DNA (cfDNA) from blood plasma using next-generation sequencing (NGS) technology. The test processes blood samples that were previously drawn using their specialized collection kit, with the blood being treated with anticoagulants to prevent clotting [21]. The comprehensive genomic analysis examines a panel of 324 genes to identify multiple genetic alterations: point mutations, insertions and deletions, structural rearrangements, and variations in gene copy numbers (both amplifications and deletions). The test also evaluates key genomic biomarkers such as blood-based tumor mutation burden (bTMB), microsatellite instability (MSI), and tumor fraction (TF) [22]. A new advanced DNA capture method allows focused sequencing of specific areas within 75 genes at extremely high depth, enabling more sensitive detection of genetic variations. The test serves primarily as a complementary diagnostic tool for non-small-cell lung cancer (NSCLC), prostate cancer, breast cancer, and ovarian cancer. A negative test outcome does not definitively rule out the presence of genomic alterations in the tumor, making follow-up tissue-based testing necessary for confirmation [21]. However, the FDA granted approval for FoundationOne Liquid CDx as a comprehensive genomic profiling test that analyzes circulating cell-free DNA (cfDNA) across multiple cancer types.

FoundationOne Heme utilizes DNA and RNA extracted from formalin-fixed, paraffin-embedded (FFPE) tumor samples, as well as from peripheral blood (PB), bone marrow aspirate (BMA), and cytology smear samples. The test employs a hybrid-capture-based next-generation sequencing approach to detect four primary categories of genomic alterations: base substitutions, insertions and deletions, copy number alterations, and rearrangements. The coding regions of 406 genes were completed via DNA sequencing, including the introns of 31 selected genes involved in rearrangements, with a median depth of ~500× unique coverage. The RNA sequences of 265 genes commonly rearranged in cancer were used to better identify known and novel gene fusions, resulting in an average of ~6.9 million unique pairs. All specimens were reviewed by a hematopathologist or anatomic pathologist to ensure specimen viability and tumor content [23].

Results of the CGP are presented as a uniform report based on criteria of Foundation Medicine in collaboration with Biopharma and other repositories and are up-to-date with the latest findings, clinical trials, and FDA approvals.

For the CGP uptake analysis, we extrapolated the data from the Croatian Cancer Registry for the tested years. We have used the total number of cancer-related deaths, as that is the largest potential pool of our targeted population of patients with metastatic disease, and we have excluded the average percentage of patients who were in subsequent lines of therapy because CGP was reimbursed only for the first line of metastatic disease and the average percentage of patients who did not complete the CGP criteria selection [24].

### 2.3. Study Population

The study encompassed all Croatian patients who underwent comprehensive genomic profiling of their tumor samples during the two-year period from 1 January 2020 through 31 December 2021. Thus, the sample size was not selected, and a power analysis was not performed before the study started. Patients were selected by multidisciplinary teams inherent to each institution and in accordance with the aforementioned inclusion criteria. Patients qualified for CGP testing met the following criteria: they had confirmed metastatic or locally advanced, inoperable stage of disease, had expected survival time of 6 months or longer, and demonstrated an ECOG performance status of 2 or better, as well as received approval from the multidisciplinary tumor board [25].

### 2.4. Endpoints

The primary endpoint was the proportion of patients with positive CGP findings in terms of having clinically relevant genomic alterations in relation to the specific tumor type.

### 2.5. Statistical Analysis

The data are presented as percentages, medians, and interquartile ranges (IQRs) or means and standard deviations. Binary logistic regression was used to analyze the statistical significance of differences in the odds of the presence of clinically relevant mutations between tumors of different affected organ systems. The false-positive rate was controlled via the Benjamini–Hochberg procedure with false discovery rate (FDR) < 5%. Statistical analysis was conducted via StataCorp 2024 software (Stata Statistical Software: Release 18.5 College Station, TX, USA: StataCorp LLC).

## 3. Results

### 3.1. Patients Characteristics

A total of 481 patients were presented at multidisciplinary teams and CGP was performed on their tumors between 1 January 2020 and 31 December 2021. There was an almost equal distribution between the sexes with 202 (42%) males and 279 (58%) females tested. The median age of the patients was 61 years (IQR 49–69). Gastrointestinal and reproductive malignancies were the most common, accounting for 29.1% and 28.9% of all tested tumors, respectively. Specifically, colorectal tumors made up 19.1% of cases, while uterine tumors represented 11.2% (Table 1).

### 3.2. Results of the CGP Analysis

Genomic testing revealed that more than three-quarters (76.7%) of patients harbored significant mutations, with KRAS mutations representing the most common alteration, occurring in 27.2% of cases (Table 2). The next most common clinically relevant alterations were those in the *phosphatidylinositol-4,5-bisphosphate 3-kinase catalytic subunit alpha* (*PIK3CA*), *androgen receptor* (*AR*), *neuroblastoma RAS viral oncogene homolog* (*NRAS*), and *phosphatase and tensin homolog* (*PTEN*) genes, which were found in 12.9%, 10.6%, 10.4%, and 9.1% of patients, respectively.

Conversely, clinically non-relevant mutations were found in 81.5% of patients, with the *tumor protein P53* (*TP53*) (42.8%) mutation being the most common (Supplementary Table S1). The next most prevalent alterations without clinical significance were those in *adenomatous polyposis coli* (*APC*), *cyclin-dependent kinase inhibitor 2A* (*CDKN2A*), *AR*, and *NRAS* genes, which were found in 15.6%, 9.8%, 8.9%, and 8.3% of patients. Compared to the gastrointestinal system, the genitourinary, respiratory, musculoskeletal, endocrine, and head and neck primary tumor sites have significantly lower odds of clinically relevant mutations (Table 3). These findings suggest that certain organ systems may have a lower prevalence of clinically relevant mutations, which could impact clinical management and the research directions in precision oncology.

### 3.3. Uptake of the CGP

According to the Croatian Cancer Registry, there were 26,709 (13,138 in 2020 and 13,571 in 2021) cancer-related deaths during the investigated two-year period; combining this information with the CGP selection criteria valid at that time, there was an estimated population of approximately 13,350 potentially eligible patients for CGP analysis, indicating that only 3.7% of potentially eligible patients were tested (Table 4).

## 4. Discussion

Undoubtedly, precision oncology is the present and future of oncology, and CGP is of the utmost importance for its applicability in everyday clinical practice. The true value of the precision oncology concept has been tested in numerous clinical trials, and many more studies are currently being conducted. Even though trials such as SHIVA or some of the cohorts from the TAPUR trial have had negative results regarding the outcomes of targeted therapies matching the genomic alterations, by addressing their limitations and obstacles, they have influenced future trial designs [26]. Moreover, other studies, such as IMPACT, IMPACT2, or other cohorts from the TAPUR study, have shown the benefits of a tailored treatment and have promoted these therapies as potentially better treatment options than conventional therapies [26]. Throughout the evolution of these trials, multiple important issues have been addressed, such as the limitations of the tumor tissue used for the analysis, the matter of which tissue should be tested, delays in the process of testing, interpretations of the results, the selection of the appropriate treatment, particularly in the case of multiple genomic alterations (single agent versus combinational treatment), the procurement of the treatment, and many others [26].

Our results concerning the distribution of clinically relevant mutations by the primary affected organ system have shown generally high odds of mutation. There are significantly lower odds of revealing clinically relevant alterations in tumors from some organ systems, such as genitourinary, endocrine, and musculoskeletal tumors. Nevertheless, the fact that the lowest odds of having clinically relevant mutations are in the range of approximately 35% indicates that, most likely, the best approach is to test all patients to prevent any patients from being underserved. Our results showed that the CGP analysis revealed at least one actionable clinically relevant alteration in nearly 80% of the tested patients, with a range of 35–88%. Despite such findings and considering that CGP has been widely available in Croatia since 2020, we could, unfortunately, state that less than 5% of potentially eligible patients were tested within the first two years. The implementation of different diagnostic and/or treatment methods in medicine is usually based on their price, complexity, availability, efficacy/impact, and level of medical system development, as well as general public education [27]. In Croatia, CGP is fully covered by Croatian health insurance, but its penetration in everyday clinical practice has been somewhat limited across the country. The administrative requirements and paperwork needed per patient were potentially the greatest problems in the efficient and effective implementation of the CGP. Furthermore, the complexity of the results and the absence of a molecular tumor board (MTB) at the time of testing could lead to a lower uptake among oncology professionals, which is similar to Italy [28]. Nevertheless, one can argue, on the basis of the analysis performed among Italian professionals, that the majority did not refer their patients to the molecular tumor board despite having access [28]. In addition, terms such as distraction and enchantment have been associated with the implementation of precision medicine in everyday clinical practice among health care professionals [18]. Although the MTB comprises different professionals, such as oncologists, pathologists, geneticists, molecular biologists, biostatisticians, and data administrators, and is indicative of interpretations of the results of the testing and exchange of knowledge, there are already guidelines for oncology specialists on how to read the reports to navigate through this wealth of information [29]. Nonetheless, there are multiple potential reasons for the low uptake rate of CGP in everyday clinical work, such as missing tumor tissue or tissue insufficiency, an administrative load with a shortage of personnel, inexperience with the testing process, and, hence, a long turnaround time, trouble with data interpretation, and drug unavailability. At the time of the analysis, Croatia had not established an MTB or a method for obtaining treatment. Drug procurement is still an unresolved barrier in many countries and one of the reasons many trials have been performed, and many patients could still be left underserved because they do not fit the trial criteria [26]. Since August 2021, Croatia has had a National Committee for CGP-guided treatment whose role is to debate and interpret the results of CGP testing and indicate the treatment in accordance with the results. Additionally, the Croatian Ministry of Health provided a significant financial fund specifically for this type of treatment, and a future analysis of the CGP-guided therapy results is eagerly awaited. The fact that we performed 54% fewer CGP tests in the second year than in the first defines more serious and structural problems we must work on to improve the situation with precision oncology implementation in Croatia.

The CGP has gradually been introduced in everyday clinical practice and has already proven its utility and applicability across different countries and tumor types [19,30,31,32]. Healthcare systems need to adapt to accommodate the complexities of precision oncology, including reimbursement policies and infrastructure development. In conclusion, while precision oncology is increasingly penetrating everyday clinical practice, its implementation remains uneven. Continued efforts in education, standardization, and healthcare system adaptation are necessary in order to ensure the equitable access to and optimal utilization of precision oncology approaches for cancer patients. Sharing the national-level experience of precision oncology implementation could be helpful in the process of optimizing its implementation.

### Study Limitations

Our study has several limitations. The retrospective study design could introduce relative imprecision in our analysis of the results. In addition, patient selection for CGP analysis by practicing oncologists could be a confounding factor that could influence our results. Therefore, future studies with larger sample sizes and diverse populations are needed in order to validate our findings and address these potential confounding factors. The relatively small sample size limits the statistical power to detect differences between particular system organs, especially in rarer tumor types, and potentially underestimates the presence of clinically relevant mutations.

## 5. Conclusions

The analysis identified clinically actionable genomic alterations in approximately 80% of the evaluated patients, suggesting they could be candidates for targeted therapeutic interventions. The adoption of CGP remains limited, with estimates indicating that under 5% of metastatic cancer patients received testing in the initial two-year implementation period, despite established national insurance coverage guidelines. This low utilization rate suggests a significant gap in access to genomic testing, leaving many eligible cancer patients without the potential benefits of this diagnostic approach. With a broader implementation of precision oncology and CGP-guided therapy, real-world data monitoring and reporting are critically important for defining its true value and optimal position in everyday clinical practice.

## Figures and Tables

**Table 1:** Distribution of CGP by diagnosis and year.

	2020 (n = 313)	2021 (n = 168)	Total (n = 481)
Diagnosed cancers by organ system			
Gastrointestinal	92 (29.4)	48 (28.6)	140 (29.1)
Reproductive organs (gynecologic and breast cancer)	92 (29.4)	47 (28.0)	139 (28.9)
Respiratory	52 (16.6)	18 (10.7)	70 (14.6)
Musculoskeletal	16 (5.1)	18 (10.7)	34 (7.1)
Genitourinary	20 (6.4)	8 (4.8)	28 (5.8)
Skin	10 (3.2)	7 (4.2)	17 (3.5)
Endocrine	8 (2.6)	4 (2.4)	12 (2.5)
Central nervous system	6 (1.9)	3 (1.8)	9 (1.9)
Head and neck	4 (1.3)	2 (1.2)	6 (1.2)
Other	13 (4.2)	13 (7.7)	26 (5.4)
Specific diagnosis, n (%)			
Colorectal	65 (20.8)	27 (16.1)	92 (19.1)
Lung	44 (14.1)	13 (7.7)	57 (11.9)
Ovaries	25 (8.0)	21 (12.5)	46 (9.6)
Endometrium	29 (9.3)	10 (6.0)	39 (8.1)
Breast	23 (7.3)	10 (6.0)	33 (6.9)
Stomach, abdominal	11 (3.5)	6 (3.6)	17 (3.5)
Skin, melanoma	9 (2.9)	7 (4.2)	16 (3.3)
Soft tissue and musculoskeletal system	8 (2.6)	8 (4.8)	16 (3.3)
Uterus	11 (3.5)	4 (2.4)	15 (3.1)
Pancreas	7 (2.2)	6 (3.6)	13 (2.7)
Kidney	7 (2.2)	3 (1.8)	10 (2.1)
Other	74 (23.6)	53 (31.5)	127 (26.4)

Data are presented as the number (percentage) of patients.

**Table 2:** Genes with clinically relevant mutations (n = 481).

	n	(%)		n	(%)		n	(%)
KRAS	131	(27.2)	PTCH1	6	(1.2)	MAP3K1	1	(0.2)
PIK3CA	62	(12.9)	PALB2	5	(1.0)	MLH1	1	(0.2)
AR	51	(10.6)	CCND2	4	(0.8)	MSH3	1	(0.2)
NRAS	50	(10.4)	GNAQ	4	(0.8)	NTRK2	1	(0.2)
PTEN	44	(9.1)	PDGFRA	4	(0.8)	NTRK3	1	(0.2)
ARID1A	43	(8.9)	ALK	3	(0.6)	PDGFRB	1	(0.2)
MYC	35	(7.3)	BAP1	3	(0.6)	PIK3CB	1	(0.2)
NF1	24	(5.0)	BRIP1	3	(0.6)	RB1	1	(0.2)
BRCA1	23	(4.8)	MEN1	3	(0.6)	SMARCA4	1	(0.2)
CTNNB1	22	(4.6)	RET	3	(0.6)	SMO	1	(0.2)
STK11	20	(4.2)	AKT3	2	(0.4)	SUFU	1	(0.2)
ATM	17	(3.5)	AXL	2	(0.4)			
ERBB2	17	(3.5)	FLT3	2	(0.4)			
CHEK2	15	(3.1)	GNA11	2	(0.4)			
PIK3R1	15	(3.1)	HRAS	2	(0.4)			
BRAF	14	(2.9)	MDM4	2	(0.4)			
CDK4	13	(2.7)	MTOR	2	(0.4)			
FBXW7	13	(2.7)	MYCN	2	(0.4)			
AURKA	11	(2.3)	PBRM1	2	(0.4)			
BRCA2	11	(2.3)	RAD54L	2	(0.4)			
EGFR	11	(2.3)	RAF1	2	(0.4)			
MDM2	11	(2.3)	ALOX12B	1	(0.2)			
NF2	10	(2.1)	APC	1	(0.2)			
RNF43	10	(2.1)	ARAF	1	(0.2)			
CCND1	8	(1.7)	ATR	1	(0.2)			
FGFR1	8	(1.7)	CBL	1	(0.2)			
MET	8	(1.7)	CCNE1	1	(0.2)			
SOX2	8	(1.7)	CDK12	1	(0.2)			
AKT1	7	(1.5)	CDK6	1	(0.2)			
AKT2	7	(1.5)	CDKN1A	1	(0.2)			
ERBB3	7	(1.5)	CDKN2A	1	(0.2)			
MTAP	7	(1.5)	EP300	1	(0.2)			
RICTOR	7	(1.5)	ERRFI1	1	(0.2)			
SMARCB1	7	(1.5)	EZH2	1	(0.2)			
FANCL	6	(1.2)	FANCA	1	(0.2)			
FGFR2	6	(1.2)	FGFR4	1	(0.2)			
IDH1	6	(1.2)	FLCN	1	(0.2)			
KEAP1	6	(1.2)	KDM6A	1	(0.2)			
KIT	6	(1.2)	MAF	1	(0.2)			

**Table 3:** Clinically relevant mutations by cancer primary affected organ system (n = 481).

	n (%)	OR	(95% CI)	*p*
Gastrointestinal	123 (87.9)	1.00	(referent)	
Genitourinary	14 (50.0)	0.14	(0.06; 0.34)	<0.001 *
Reproductive organs	119 (85.6)	0.82	(0.41; 1.65)	0.581
Respiratory	47 (67.1)	0.28	(0.14; 0.58)	<0.001 *
Musculoskeletal	12 (35.3)	0.08	(0.03; 0.18)	<0.001 *
Skin	15 (88.2)	1.04	(0.22; 4.93)	0.964
Endocrine	6 (50.0)	0.14	(0.04; 0.48)	0.002 *
Central nervous system	8 (88.9)	1.11	(0.13; 9.40)	0.927
Head and neck	3 (50.0)	0.14	(0.03; 0.74)	0.021 *
Other	22 (84.6)	0.76	(0.23; 2.47)	0.649

Abbreviations: OR, odds ratio; CI, confidence interval; *p*, statistical significance of the OR calculated via binary logistic regression. * FDR < 5%.

**Table 4:** Distribution of potential CGP candidates and actual CGP implementation across different cancer organ systems.

	Candidates *	CGP	Percent Tested
Gastrointestinal	4499 (35.0)	140 (29.1)	3.1%
Genitourinary	1883 (14.6)	28 (5.8)	1.5%
Reproductive organs	1333 (10.4)	139 (28.9)	10.4%
Respiratory	2951 (22.9)	70 (14.6)	2.4%
Musculoskeletal	90 (0.7)	34 (7.1)	37.8%
Skin	224 (1.7)	17 (3.5)	7.6%
Endocrine	34 (0.3)	12 (2.5)	35.3%
Central nervous system	391 (3.0)	9 (1.9)	2.3%
Head and neck	488 (3.8)	6 (1.2)	1.2%
Other	973 (7.6)	26 (5.4)	2.7%
TOTAL	12,866 (100.0)	481	3.7%

Data are presented as the number (percentage) of patients if not stated otherwise. Abbreviations: CGP, comprehensive genomic profiling. * The number of candidates for CGP was estimated based on the GLOBOCAN 2022 mortality data; this number was multiplied by two to account for the two-year duration of our study, and then divided by 0.50 (explained in the methods).

## Data Availability

The original contributions presented in this study are included in the article/Supplementary Materials. Further inquiries can be directed to the corresponding authors.

## Supplementary Materials

The following supporting information can be downloaded at: https://www.mdpi.com/article/10.3390/jpm15020059/s1, Table S1: Genes with clinically non relevant mutations (n = 481).

## Abbreviations

The following abbreviations are used in this manuscript:

APC	adenomatous polyposis coli
CDKN2A	cyclin-dependent kinase inhibitor 2A
cfDNA	cell-free DNA
CGP	comprehensive genomic profiling
CI	confidence interval
CNA	copy number alteration
ECOG PS	Eastern Cooperative Oncology Group performance status
FDA	U.S. Food and Drug Administration
FDR	false discovery rate
IQR	interquartile range
MSI	microsatellite instability
MTB	Molecular Tumor Board
Muts/Mb	mutations per megabase
NSCLC	non-small cell lung cancer
OR	odds ratio
TF	tumor fraction
TMB	tumor mutational burden
TP53	tumor protein P53
Abbreviations from Table 2	
KRAS	Kirsten rat sarcoma
PIK3CA	phosphatidylinositol-4,5-bisphosphate 3-kinase catalytic subunit alpha
AR	androgen receptor
NRAS	neuroblastoma RAS viral oncogene homolog
PTEN	phosphatase and tensin homolog
ARID1A	AT-rich interactive domain-containing protein 1A
MYC	Myelocytomatosis oncogene
NF1	Neurofibromatosis type 1
BRCA1	Breast cancer 1, early onset
CTNNB1	Catenin beta 1
STK11	Serine/threonine kinase 11
ATM	Ataxia telangiectasia mutated
ERBB2	Erb-b2 receptor tyrosine kinase 2
CHEK2	Checkpoint kinase 2
PIK3R1	Phosphoinositide-3-kinase regulatory subunit 1
BRAF	B-Raf proto-oncogene, serine/threonine kinase
CDK4	Cyclin-dependent kinase 4
FBXW7	F-box and WD repeat domain containing 7
AURKA	Aurora kinase A
BRCA2	Breast cancer 2, early onset
EGFR	Epidermal growth factor receptor
MDM2	Mouse double minute 2 homolog
NF2	Neurofibromatosis type 2
RNF43	Ring finger protein 43
CCND1	Cyclin D1
FGFR1	Fibroblast growth factor receptor 1
MET	Mesenchymal epithelial transition factor
SOX2	SRY-box transcription factor 2
AKT1	AKT serine/threonine kinase 1
AKT2	AKT serine/threonine kinase 2
ERBB3	Erb-b2 receptor tyrosine kinase 3
MTAP	Methylthioadenosine phosphorylase
RICTOR	Rapamycin-insensitive companion of mTOR
SMARCB1	SWI/SNF-related, matrix-associated, actin-dependent regulator of chromatin subfamily B member 1
FANCL	Fanconi anemia complementation group L
FGFR2	Fibroblast growth factor receptor 2
IDH1	Isocitrate dehydrogenase (NADP(+)) 1, cytosolic
KEAP1	Kelch-like ECH-associated protein 1
KIT	KIT proto-oncogene, receptor tyrosine kinase
PTCH1	Patched 1
PALB2	Partner and localizer of BRCA2
CCND2	Cyclin D2
GNAQ	G protein subunit alpha q
PDGFRA	Platelet-derived growth factor receptor alpha
ALK	Anaplastic lymphoma kinase
BAP1	BRCA1 associated protein 1
BRIP1	BRCA1 interacting protein C-terminal helicase 1
MEN1	Multiple endocrine neoplasia type 1
RET	Ret proto-oncogene
AKT3	AKT serine/threonine kinase 3
AXL	AXL receptor tyrosine kinase
FLT3	FMS-like tyrosine kinase 3
GNA11	G protein subunit alpha 11
HRAS	Harvey rat sarcoma viral oncogene homolog
MDM4	Mouse double minute 4 homolog
MTOR	Mechanistic target of rapamycin
MYCN	V-myc avian myelocytomatosis viral oncogene neuroblastoma derived homolog
PBRM1	Polybromo 1
RAD54L	RAD54 like
RAF1	RAF proto-oncogene serine/threonine-protein kinase
ALOX12B	Arachidonate lipoxygenase 12B
APC	Adenomatous polyposis coli
ARAF	ARAF proto-oncogene, serine/threonine kinase
ATR	ATR serine/threonine kinase
CBL	Casitas B-lineage lymphoma proto-oncogene
CCNE1	Cyclin E1
CDK12	Cyclin-dependent kinase 12
CDK6	Cyclin-dependent kinase 6
CDKN1A	Cyclin-dependent kinase inhibitor 1A (p21)
CDKN2A	Cyclin-dependent kinase inhibitor 2A (p16)
EP300	E1A binding protein p300
ERRFI1	ERBB receptor feedback inhibitor 1
EZH2	Enhancer of zeste homolog 2
FANCA	Fanconi anemia complementation group A
FGFR4	Fibroblast Growth Factor Receptor 4
FLCN	Folliculin
KDM6A	Lysine Demethylase 6A
MAF	MAF Proto-Oncogene
MAP3K1	Mitogen-Activated Protein Kinase Kinase Kinase 1
MLH1	MutL Homolog 1
MSH3	MutS Homolog 3
NTRK2	Neurotrophic Tyrosine Receptor Kinase 2
NTRK3	Neurotrophic Tyrosine Receptor Kinase 3
PDGFRB	Platelet-Derived Growth Factor Receptor Beta
PIK3CB	Phosphoinositide 3-Kinase Catalytic Subunit Beta
RB1	Retinoblastoma 1
SMARCA4	SWI/SNF Related, Matrix Associated, Actin Dependent Regulator of Chromatin Subfamily A Member 4
SMO	Smoothened, Frizzled Class Receptor
SUFU	Suppressor of Fused
