# Supplementary material for: Precision Oncology in Clinical Practice: Two Years of Comprehensive Genomic Profiling in Croatia
Source: J Pers Med. 2025 Jan 31;15(2):59. doi: 10.3390/jpm15020059 (PMC11856208; doi:10.3390/jpm15020059)
Supplement: Supplementary file 1 [file jpm-15-00059-s001.zip › jpm-3440460-supplementary.pdf]

SUPPLEMENTARY TABLE

Supplementary Table S1. Genes with clinically non relevant mutations (n = 481)

	n	(%)
TP53	206	(42.8)
APC	75	(15.6)
CDKN2A	47	(9.8)
AR	43	(8.9)
NRAS	40	(8.3)
RB1	35	(7.3)
RAD21	23	(4.8)
DNMT3A	18	(3.7)
GNAS	18	(3.7)
BCOR	17	(3.5)
CCNE1	17	(3.5)
SMAD4	17	(3.5)
ATR	16	(3.3)
CDKN2B	16	(3.3)
KRAS	16	(3.3)
MTAP	14	(2.9)
NF2	14	(2.9)
ATRX	13	(2.7)
SMARCA4	13	(2.7)
NFKBIA	12	(2.5)
ZNF217	12	(2.5)
BAP1	11	(2.3)
BCL2	11	(2.3)
BRD4	11	(2.3)
NOTCH3	11	(2.3)
ARFRP1	10	(2.1)
BCL2L1	10	(2.1)
CIC	10	(2.1)
PBRM1	10	(2.1)
SOX9	10	(2.1)
MSH3	9	(1.9)
MCL1	8	(1.7)
CHEK2	7	(1.5)
CREBBP	7	(1.5)
FGF19	7	(1.5)
IRS2	7	(1.5)
KDM5C	7	(1.5)
MAP3K1	7	(1.5)
NKX2-1	7	(1.5)
PIK3C2B	7	(1.5)
BCORL1	6	(1.2)
BRAF	6	(1.2)
CDH1	6	(1.2)
EP300	6	(1.2)
FGF12	6	(1.2)
FGF3	6	(1.2)

	n	(%)
FGF4	6	(1.2)
JUN	6	(1.2)
KEL	6	(1.2)
MAP2K4	6	(1.2)
MDM4	6	(1.2)
MYC	6	(1.2)
NBN	6	(1.2)
PIK3CA	6	(1.2)
PRKCI	6	(1.2)
SETD2	6	(1.2)
U2AF1	6	(1.2)
VHL	6	(1.2)
WHSC1	6	(1.2)
ACVR1B	5	(1.0)
ARID1A	5	(1.0)
ASXL1	5	(1.0)
CTCF	5	(1.0)
CTNNB1	5	(1.0)
EPHB1	5	(1.0)
ERBB4	5	(1.0)
ESR1	5	(1.0)
FGF14	5	(1.0)
MSH6	5	(1.0)
MUTYH	5	(1.0)
NOTCH1	5	(1.0)
STAG2	5	(1.0)
STK11	5	(1.0)
CASP8	4	(0.8)
CDK8	4	(0.8)
CTNNA1	4	(0.8)
ERBB3	4	(0.8)
FGF10	4	(0.8)
FGF23	4	(0.8)
FGF6	4	(0.8)
IKBKE	4	(0.8)
JAK1	4	(0.8)
KDM5A	4	(0.8)
KDM6A	4	(0.8)
KDR	4	(0.8)
MLH1	4	(0.8)
PTEN	4	(0.8)
PTPRO	4	(0.8)
QKI	4	(0.8)
SMAD2	4	(0.8)
SPEN	4	(0.8)
SRC	4	(0.8)
TET2	4	(0.8)
TGFBR2	4	(0.8)
CBFB	3	(0.6)
CDC73	3	(0.6)

	n	(%)
CUL4A	3	(0.6)
DIS3	3	(0.6)
FANCC	3	(0.6)
FLCN	3	(0.6)
INPP4B	3	(0.6)
JAK2	3	(0.6)
MRE11A	3	(0.6)
NTRK1	3	(0.6)
PPP2R1A	3	(0.6)
PTPN11	3	(0.6)
ZNF703	3	(0.6)
AKT1	2	(0.4)
ALOX12B	2	(0.4)
ATM	2	(0.4)
CBL	2	(0.4)
CCND3	2	(0.4)
CD22	2	(0.4)
CDKN1B	2	(0.4)
CSF1R	2	(0.4)
DDR1	2	(0.4)
FAS	2	(0.4)
FH	2	(0.4)
FLT1	2	(0.4)
FLT3	2	(0.4)
FUBP1	2	(0.4)
GATA3	2	(0.4)
GATA6	2	(0.4)
GNAQ	2	(0.4)
IDH2	2	(0.4)
IGF1R	2	(0.4)
KEAP1	2	(0.4)
KIT	2	(0.4)
LYN	2	(0.4)
MDM2	2	(0.4)
MEN1	2	(0.4)
MSH2	2	(0.4)
MST1R	2	(0.4)
MYCN	2	(0.4)
NFE2L2	2	(0.4)
NOTCH2	2	(0.4)
PARK2	2	(0.4)
PDGFRA	2	(0.4)
PIK3R1	2	(0.4)
PRDM1	2	(0.4)
RBM10	2	(0.4)
SF3B1	2	(0.4)
TEK	2	(0.4)
TMPRSS2	2	(0.4)
VEGFA	2	(0.4)
AKT3	1	(0.2)

	n	(%)
AURKB	1	(0.2)
AXIN1	1	(0.2)
AXL	1	(0.2)
CARD11	1	(0.2)
CCND1	1	(0.2)
CD70	1	(0.2)
CD79A	1	(0.2)
CDK12	1	(0.2)
CDK4	1	(0.2)
CDKN1A	1	(0.2)
CEBPA	1	(0.2)
CRKL	1	(0.2)
CSF3R	1	(0.2)
CUL3	1	(0.2)
DAXX	1	(0.2)
DDR2	1	(0.2)
EED	1	(0.2)
EPHA3	1	(0.2)
EPHB4	1	(0.2)
ERBB2	1	(0.2)
ERCC4	1	(0.2)
ERRFI1	1	(0.2)
FGFR1	1	(0.2)
FOXL2	1	(0.2)
GRM3	1	(0.2)
HGF	1	(0.2)
HNF1A	1	(0.2)
IKZF1	1	(0.2)
IRF2	1	(0.2)
MED12	1	(0.2)
MITF	1	(0.2)
NF1	1	(0.2)
PALB2	1	(0.2)
PIK3C2G	1	(0.2)
PIM1	1	(0.2)
POLD1	1	(0.2)
PPP2R2A	1	(0.2)
PRKAR1A	1	(0.2)
PTCH1	1	(0.2)
RAD51	1	(0.2)
RAF1	1	(0.2)
RARA	1	(0.2)
REL	1	(0.2)
RICTOR	1	(0.2)
RNF43	1	(0.2)
ROS1	1	(0.2)
SDHB	1	(0.2)
SGK1	1	(0.2)
SMO	1	(0.2)
SOX2	1	(0.2)

	n	(%)
STAT3	1	(0.2)
SUFU	1	(0.2)
TBX3	1	(0.2)
TNFAIP3	1	(0.2)
TSC2	1	(0.2)
WT1	1	(0.2)
